# Assessment of bone mineral density by dual energy x-ray absorptiometry in patients with mucopolysaccharidoses

**DOI:** 10.1186/1750-1172-8-71

**Published:** 2013-05-11

**Authors:** Hsiang-Yu Lin, Shou-Chuan Shih, Chih-Kuang Chuang, Ming-Ren Chen, Dau-Ming Niu, Shuan-Pei Lin

**Affiliations:** 1 Department of Medicine, Mackay Medical College, New Taipei City, Taiwan; 2 Department of Pediatrics, Mackay Memorial Hospital, No.92, Sec. 2, Chung-Shan North Road, Taipei 10449, Taiwan; 3 Department of Medical Research, Mackay Memorial Hospital, Taipei, Taiwan; 4 Department of Early Infant Care and Education, Mackay Medicine, Nursing and Management College, Taipei, Taiwan; 5 Institute of Clinical Medicine, National Yang-Ming University, Taipei, Taiwan; 6 Division of Gastroenterology, Department of Internal Medicine, Mackay Memorial Hospital, Taipei, Taiwan; 7 Medical College, Fu-Jen Catholic University, Taipei, Taiwan; 8 Institute of Biotechnology, National Taipei University of Technology, Taipei, Taiwan; 9 Department of Pediatrics, Taipei Veterans General Hospital, Taipei, Taiwan; 10 Department of Infant and Child Care, National Taipei University of Nursing and Health Sciences, Taipei, Taiwan

**Keywords:** Bone mineral density, Dual energy x-ray absorptiometry, Enzyme replacement therapy, Mucopolysaccharidoses

## Abstract

**Background:**

Patients with mucopolysaccharidoses (MPS) are associated with poor bone growth and mineralization, however, information regarding the assessment of bone mineral density (BMD) in relation to age and treatment in this disorder is limited.

**Methods:**

Dual energy x-ray absorptiometry (DXA) was performed in 30 patients with MPS (21 males and 9 females; 2 with MPS I, 12 with MPS II, 2 with MPS IIIB, 9 with MPS IVA, and 5 with MPS VI; median age, 10.8 years; age range, 5.0 years to 23.7 years; 26 patients were under 19 and 4 were above 19 years of age) to assess BMD of the lumbar spine (L1-L4), using the Hologic QDR 4500 system (Bedford, MA, USA).

**Results:**

For 26 patients under 19 years of age, standard deviation scores (z scores) for height, weight, body mass index (BMI), and BMD were −4.53 ± 2.66, -1.15 ± 1.55, 0.74 ± 1.23, and −3.03 ± 1.62, respectively, and they were all negatively correlated with age (*p* < 0.05). However, after correction for height-for-age z score (HAZ), HAZ adjusted BMD z score was −0.7 ± 1.24. Eight patients (31%) had osteopenia (HAZ adjusted BMD z score < −1 and ≥ −2), and 4 patients (15%) had osteoporosis (HAZ adjusted BMD z score < −2). Of 8 patients with MPS I, II or VI who underwent follow-up DXA after receiving enzyme replacement therapy for 1.0 to 7.4 years, all showed increase in absolute BMD values.

**Conclusions:**

These findings and the follow-up data can be used to develop quality of care strategies for patients with MPS.

## Introduction

The mucopolysaccharidoses (MPS; OMIM 252700) are a group of inherited lysosomal storage disorders caused by deficiencies in enzymes catalyzing the degradation of glycosaminoglycans (GAGs). Progressive lysosomal accumulation of GAGs results in profound growth deficits, skeletal deformities (dysostosis multiplex), poor joint mobility, coarse facial features and organomegaly. Eleven known enzyme deficiencies give rise to 7 distinct types of MPS (I, II, III, IV, VI, VII, and IX). All are inherited in an autosomal recessive manner except for MPS II, which is an X-linked recessive disorder that occurs almost exclusively in males. Each MPS type exhibits a wide spectrum of clinical severity. Both severe and attenuated forms exist for MPS I (Hurler, Hurler-Scheie and Scheie syndromes) and MPS II (Hunter syndrome), and additional subtypes have been described for MPS III and MPS IV [1,2]. The prevalence of MPS as a group is reported to be 1.9-4.5/100,000 live births with geographical differences in the frequencies of specific types. In Taiwan and other Asian countries, the most common type is MPS II, whereas in most Caucasian countries, it is MPS I or MPS III [3].

It is well-known that many children with chronic illnesses are at risk for low bone mass [4-9]. Patients with MPS have an increased risk of poor bone mineralization due to malnutrition, a particularly small frame, an abnormal gait, and reduction of physical activities caused by pain, poor health condition, or exercise intolerance [10]. Although bone growth and mineralization have been reported to be affected by GAGs accumulation in animal models of MPS [11,12], there is little published literature on the assessment of bone mineral density (BMD) in patients with MPS [10,13,14]. Dual energy x-ray absorptiometry (DXA) is the most frequently used tool to evaluate BMD because of its low radiation exposure and rapid scan time. Fung et al. [10] evaluated BMD by DXA in 8 patients with MPS II or VI who were receiving enzyme replacement therapy (ERT) and found that BMD was within the normal range for most of the patients, particularly after correction for short stature. However, BMD was not measured prior to the initiation of ERT, and thus, it is unclear whether the normal values existed prior to ERT or represented a treatment effect. Here, we characterized BMD and growth deficits in a group of 30 MPS patients not selected for skeletal problems and before the initiation of ERT. We hypothesized that MPS patients would have abnormally low BMD z scores, which has never been formally studied. Therefore we evaluated BMD by DXA in patients with MPS, in relation to age and treatment with ERT.

## Materials and methods

### Subjects

Thirty patients with MPS (21 males and 9 females; median age, 10.8 years; age range, 5–23.7 years; 26 patients were under age 19 and 4 patients above age 19) were enrolled in this study at Mackay Memorial Hospital, Taipei, Taiwan. All patients underwent a history taking for skeletal problems, a physical examination to determine Tanner staging, and DXA. None had received ERT, hematopoietic stem cell transplantation or medications that might affect BMD prior to their baseline DXA. The diagnosis of MPS was confirmed by two-dimensional electrophoresis of urinary GAGs and enzyme activity assays in serum, leukocytes and/or skin fibroblasts. The study population consisted of 2 patients with MPS I (Hurler-Scheie), 12 with MPS II, 2 with MPS IIIB, 9 with MPS IVA, and 5 with MPS VI [15]. Written informed consent was obtained from a parent for children and from patients over 18 years. The study was approved by the ethics committee of Mackay Memorial Hospital.

### Assessments

Each patient’s gender, age, MPS type, height, weight, and body mass index (BMI) were recorded at baseline. Puberty was defined as Tanner stage ≥3 by physical examination. A standard deviation score (z score) was derived by subtracting the population mean from each individual’s raw score and then dividing the difference by the standard deviation of the population. Z scores for height, weight, and BMI were calculated based on standard growth tables for Taiwanese children [16]. Results are expressed as the mean ± standard deviation unless otherwise indicated. BMD of the lumbar spine (L1-L4) was assessed by DXA using the Hologic QDR 4500 system (Hologic, Bedford, MA, USA) [17]. For the 26 patients under 19 years of age, the BMD results were converted to age- and gender-specific z scores based on the normative reference data for BMD in Taiwanese children [18]. Due to the considerable height deficits for MPS patients, BMD z scores were then adjusted for height-for-age z score (HAZ) according to the method of Zemel et al. [19] which provides an adjustment for growth deficits in BMD by DXA. According to the classification used by our radiologists, osteopenia was defined as a HAZ adjusted BMD z score between −1 and −2 (<−1 and ≥ −2) and osteoporosis as a HAZ adjusted BMD z score < −2. For the 4 patients above age 19, the BMD results were converted to gender-specific T-scores based on normative reference data for BMD in the young adult Taiwanese population [20]. The World Health Organization (WHO) defines osteoporosis in adults as a lumbar spine DXA T-score < −2.5.

### Statistical analysis

The relationship between age and various physical characteristics of the 26 MPS patients under 19 years of age was determined using Pearson’s correlation coefficient (r), and significance was tested using Fisher’s *r-z* transformations. All statistical analyses were performed using SPSS version 11.5 (SPSS Inc., Chicago, IL, USA), and differences with *p* < 0.05 were considered statistically significant.

## Results

The 30 MPS patients (21 males, 9 females, all Asian) were evenly distributed by age from 5 years to early adulthood. The excess of males was attributable to the 12 male patients with MPS II, an X-linked recessive disorder. By medical history, there was a high prevalence of musculoskeletal disease, with 13 (43%) patients having skeletal pain, 30 (100%) joint stiffness, 20 (67%) kyphosis/gibbus, 14 (47%) scoliosis, 25 (83%) claw hands, 28 (93%) vertebral deformities, 14 (47%) vertebral instability, and 13 (43%) spinal cord compression.

**Figure 1:**
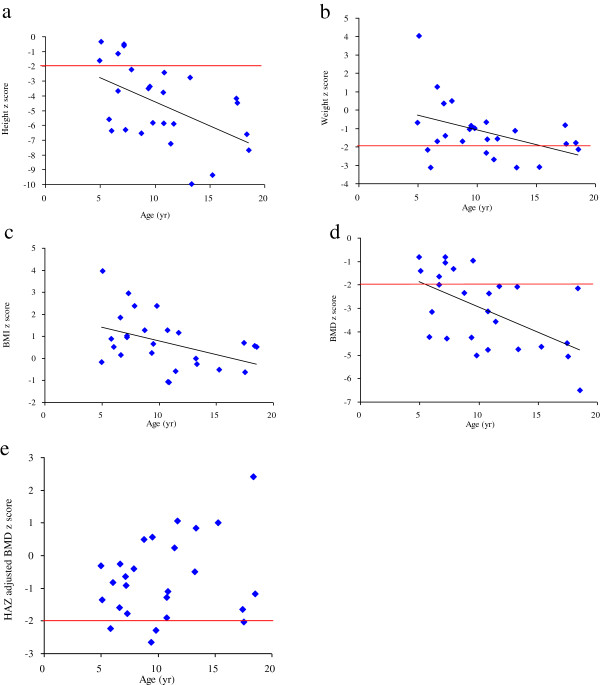
**Age against 5 parameters of 26 patients <19 years of age.** The 5 parameters were standard deviation scores (z scores) for height (**1a**), weight (**1b**), body mass index (BMI) (**1c**), bone mineral density (BMD) (**1d**), and height-for-age z scores (HAZ) adjusted BMD (**1e**). The values of the former 4 parameters (**1a-1d)** all decreased with age (*p* < 0.05). Horizontal lines represent the lower limits of normal.

Table 1 shows the mean z scores for height, weight, BMI, BMD, and HAZ-adjusted BMD by MPS type for the 26 patients <19 years of age. Similar results with T-scores are presented in Table 2 for the 4 patients ≥19 years of age. In patients <19 years of age, the mean z scores were below the normal range for height (−4.53 ± 2.66) and BMD (−3.03 ± 1.62), and within the normal range for weight (−1.15 ± 1.55) and BMI (0.74 ± 1.23). Correspondingly, most patients were below the normal range (<−2 z score) for height (81%) and BMD (69%), while only a few were below the normal range for weight (31%) and none for BMI. All four parameters were negatively correlated with age (*r* = −0.422 to −0.553, *p* <0.05) (Table 3 and Figures 1a-d). However, after adjusting for height, the mean HAZ-adjusted BMD z score increased into the normal range (−0.7 ± 1.24). Eight patients (31%) had osteopenia (HAZ-adjusted BMD z score < −1 and ≥ −2), and 4 patients (15%) had osteoporosis (HAZ adjusted BMD z score < −2). Unlike the previous parameters, there was no correlation between HAZ-adjusted BMD z score (Figure 1e).

**Table 1:** Patient demographic and baseline characteristics in 26 patients <19 years of age

**MPS type**	**n**	**Age (yr)**	**Age range (yr)**	**Height z score**	**Weight z score**	**BMI z score**	**BMD z score**	**HAZ adjusted BMD z score**
MPS I (H/S*)	1	18.5	18.5	−7.69	−2.13	0.53	−6.51	−1.18
MPS II	10	11.1 (5.2)	5-18.4	−3.05 (1.84)	−0.19 (1.76)	1.06 (1.37)	−2.34 (1.45)	−0.61 (1.32)
MPS IIIB	2	7.2 (0)	7.2	−0.56 (0.06)	0.36 (0)	1 (0.05)	−0.93 (0.17)	−0.78 (0.20)
MPS IVA	9	10.1 (3.2)	5.8-15.3	−6.54 (2.21)	−2.27 (0.81)	0.36 (1.47)	−4.08 (0.88)	−0.89 (1.29)
MPS VI	4	9.1 (2.1)	6.6-11.7	−4.90 (1.52)	−1.50 (0.31)	0.72 (0.59)	−2.57 (1.15)	−0.33 (1.63)
Total	26	10.4 (4.2)	5-18.5	−4.53 (2.66)	−1.15 (1.55)	0.74 (1.23)	−3.03 (1.62)	−0.70 (1.24)

Values are displayed as mean (standard deviation).

*MPS*, mucopolysaccharidoses; H/S*, Hurler/Scheie; *z score*, standard deviation score; *BMI*, body mass index; *BMD*, bone mineral density; *HAZ*, height-for-age z score.

**Table 2:** Patient demographic and baseline characteristics in 4 patients ≥19 years of age

**MPS type**	**n**	**Age (yr)**	**Age range (yr)**	**Height z score**	**Weight z score**	**BMI z score**	**BMD (g/cm**^**2**^**)**	**BMD T-score**
MPS I (H/S*)	1	22.5	22.5	−6.38	−3.31	−1.58	0.614	−3.49
MPS II	2	22.6 (1.6)	21.5-23.7	−9.15 (0.33)	−2.54 (0.12)	0.47 (0.1)	0.64 (0.006)	−6.6 (0.07)
MPS VI	1	22.8	22.8	−8.88	−3.25	−0.11	0.712	−2.82
Total	4	22.6 (0.9)	21.5-23.7	−8.39 (1.36)	−2.91 (0.43)	−0.19 (0.97)	0.65 (0.04)	−4.88 (2.01)

Values are displayed as mean (standard deviation).

*MPS*, mucopolysaccharidoses; H/S*, Hurler/Scheie; *z score*, standard deviation score; *BMI*, body mass index; *BMD*, bone mineral density; *HAZ*, height-for-age z score.

**Table 3:** Correlation matrix for the relationships between age and 5 parameters for 26 patients <19 years of age

	**Height**	**Weight**	**BMI**	**BMD**	**HAZ adjusted BMD**
	**z score**	**z score**	**z score**	**z score**	**z score**
Age (n = 26)	−0.511^**^	−0.431^*^	−0.422^*^	−0.553^**^	0.262

*p < 0.05, **p < 0.01. Relationships were tested using Pearson’s r with Fisher’s r-z transformations (2-tailed).

*z score*, standard deviation score; *BMI*, body mass index; *BMD*, bone mineral density; *HAZ*, height-for-age z score.

In the 4 patients ≥19 years of age (Table 2), the pattern of characteristics was similar, but overall more severe than in the younger age group, with height (−8.39 ± 1.36 z score) and BMD (−4.88 ± 2.01 T-score) being relatively more affected than weight (−2.91 ± 0.43 z score) and BMI (−0.19 ± 0.97).

Eight patients with MPS I (n = 1), II (n = 3) and VI (n = 4) underwent follow-up DXA after receiving ERT for a mean of 4.3 ± 1.9 years (range 1.0 to 7.4 years), including 5 who were pre-pubertal and 3 post-pubertal at the start of ERT (Table 4). All 8 patients showed an increase in absolute BMD values, and 3 of the 5 patients who initiated ERT prior to puberty showed an improvement in HAZ-adjusted BMD z score (Table 4).

**Table 4:** Baseline and follow-up data of the various parameters for 8 MPS patients underwent ERT

**No.**	**MPS type**	**Gender**	**DXA**	**Age (yr)**	**ERT duration (yr)**	**Height z score**	**Weight z score**	**BMI z score**	**BMD (g/cm**^**2**^**)**	**BMD z score**	**HAZ adjusted BMD z score**
The initiation of ERT at pre-pubertal age											
1	MPS II (M*)	M	Baseline	5	2.5	−1.63	−0.69	−0.17	0.53	−0.81	−0.31
			Follow-up	7.5		−1.22	−0.77	−0.39	0.561	−1.48	−1.01
2	MPS VI	M	Baseline	6.6	3.8	−3.69	−1.69	0.17	0.496	−1.65	−0.26
			Follow-up	10.4		−5.92	−1.82	0.11	0.51	−2.75	0.09
3	MPS VI	M	Baseline	8.7	5.1	−6.52	−1.69	1.29	0.442	−2.35	0.49
			Follow-up	13.8		−7.83	−2.55	0.16	0.492	−3.20	1.25
4	MPS VI	F	Baseline	11.7	7.4	−5.88	−1.57	1.18	0.65	−2.05	1.07
			Follow-up	19.1		−8.7	−2.23	2.06	0.671	−3.10	2.83
5	MPS II (M*)	M	Baseline	13.2	4.5	−2.77	−1.12	0.00	0.636	−2.07	−0.50
			Follow-up	17.7		−3.2	−0.68	0.51	0.867	−3.99	−1.76
The initiation of ERT at post-pubertal age											
6	MPS II (M*)	M	Baseline	17.5	1.0	−4.47	−1.84	−0.62	0.774	−5.06	−2.02
			Follow-up	18.5		−3.97	−1.62	−0.49	0.796	−4.80	−1.99
7	MPS I (H/S**)	M	Baseline	18.5	5.3	−7.69	−2.13	0.53	0.648	−6.51	−1.18
			Follow-up	23.8		−7.23	−1.77	1	0.769	−5.11	NA
8	MPS VI	F	Baseline	22.8	4.5	−8.88	−3.25	−0.11	0.712	−2.82	NA
			Follow-up	27.3		−9.77	−3.14	0.81	0.789	−2.3	NA

*MPS*, mucopolysaccharidoses; M*, mild form; H/S**, Hurler/Scheie; *DXA*, dual energy x-ray absorptiometry; *ERT*, enzyme replacement therapy; *z score*, standard deviation score; *BMI*, body mass index; *BMD*, bone mineral density; *HAZ*, height-for-age z score; *NA*, not available.

## Discussion

To the best of our knowledge, this is the largest single center study of patients with MPS evaluated for BMD using DXA prior to the initiation of ERT. We found that 46% (12/26) of the MPS study patients <19 years of age had HAZ adjusted BMD scores that met our criteria for osteopenia (z score ≥ −2 to < −1) or osteoporosis (z score < −2). The older patients had even lower z scores for height, weight and BMI, illustrating the progressive nature of the disease. DXA has been applied extensively to detect osteoporosis in postmenopausal women. BMD assessed by DXA is used to calculate a T-score (standard deviation score compared with the mean peak bone mass of normal young adults), which correlates with fracture risk in this population. With increasing awareness of osteoporosis in other populations, DXA has been extended for use in children. However, the interpretation of pediatric DXA results is complicated by ongoing bone growth and bone mineral accrual [21]. Thus z scores based on age, gender, and race-specific reference data are required to categorize BMD values into normal or low density for chronological age (z score < −2). Furthermore, it is a major challenge to accurately interpret DXA results when accessing bone health in MPS patients, given their considerable height deficits, relatively thick skulls, and disproportionately large heads (macrocephaly). DXA-derived BMD is a 2-dimentional image or an “areal” (g/cm^2^) density rather than a true volumetric density (vBMD). Thus, smaller bones have lower BMD than larger bones, even with the same vBMD [22]. As a result, for a child with severe short stature, like MPS, the BMD z score will probably be overdiagnosis of osteoporosis [21]. Additionally, whole body BMD results will be overestimated if the skull bones are abnormally thickened and the head is relatively large. To reduce these estimation biases, we chose to measure BMD of the lumbar spine (L1-L4) and to calculate z scores using age, gender, and ethnicity-specific reference data [18], and adjusting for HAZ according to the method of Zemel et al. [19] for patients under 19 years of age.

**Figure 2:**
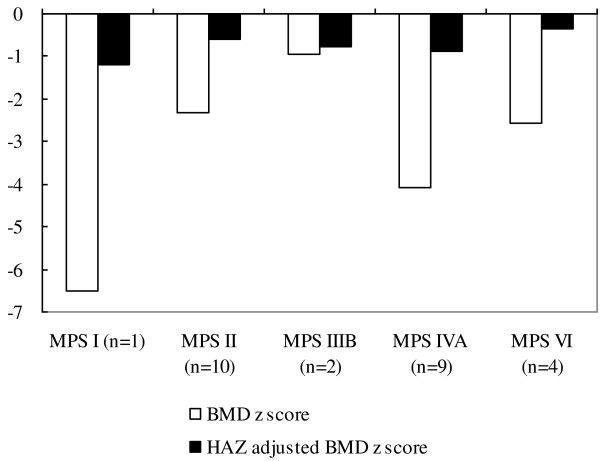
**The comparison between bone mineral density (BMD) standard deviation score (z score) and that corrected for height-for-age z score (HAZ).** Lumbar spine (L1-L4) BMD z score by dual energy x-ray absorptiometry (DXA) for chronological age (white bars) and that corrected for HAZ (black bars) in 26 patients with MPS I, II, IIIB, IVA and VI under 19 years of age.

In 2004, Rigante et al. [13] reported DXA-derived lumbar spine BMD for 3 patients with MPS III between 11 to 24 years of age. The BMD z scores of the 2 older non-weight bearing patients were lower than those of the youngest patient who could walk with support. Two years later in 2006, the same group reported normal lumbar spine BMD by DXA in 2 pre-pubertal children with MPS IV [14]. The authors suggested that the skeletal defects probably resulted from nutritional deficiencies and an inability to walk, rather than from the genetic defect itself. However, the BMD z scores in these studies were not corrected for height deficits. Since our MPS study patients had significant height deficits (height z score = −4.53 ± 2.66), we compared the unadjusted and HAZ adjusted BMD z scores for these 26 subjects under 19 years of age (Figures 1d, 1e and 2). Before adjustment, 5 patients (19%) would have met the BMD criterion for osteopenia and 18 patients (69%) met the BMD criterion for osteoporosis. However, after correcting for HAZ, the number of patients with osteopenia increased to 8 (31%), while the number of patients with osteoporosis decreased to 4 (15%). For the 4 adult patients above 19 years of age, we provided only absolute BMD values and T-scores due to the unavailability of prediction equations for adult BMD z scores adjusted for HAZ (Tables 2 and 4). As with the aforementioned concerns, since the significant height deficits of these 4 adult patients (height z score = −8.39 ± 1.36) appeared to affect the T-scores, we did not interpret the results as indicative of osteopenia or osteoporosis.

The pathophysiologic basis for skeletal findings in the lysosomal storage disorders is not completely understood. In Gaucher disease, substantial infiltration of Gaucher cells in the bone marrow, osteosclerosis, and osteonecrosis all contribute to the clinical manifestations of bone pain, bone crises, osteopenia, pathologic fractures, and avascular necrosis of the femoral head [22,23]. Similarly, in Fabry disease [24,25], Pompe disease [4], and Niemann-Pick disease type B [5], osteopenia and osteoporosis have been reported as common skeletal manifestations. ERT is an effective treatment for the systemic manifestations of Gaucher disease, and it can have a significant impact on skeletal manifestations [26-28]. ERT also has been shown to alleviate morbidity and improve endurance in MPS I, II, and VI [29-34], but there is a paucity of literature describing the effects of ERT on BMD in MPS [10,32]. Fung et al. [10] assessed vBMD in 6 patients with MPS VI before and after ERT. The authors found an inverse relationship between change in spine vBMD z score and change in height, suggesting that the BMD z score may decrease as a patient’s growth improves on ERT. Lin et al. [32] reported on an 18 year-old patient with MPS I whose BMD improved significantly after receiving ERT for 2 years. In our present study, all 8 patients with MPS I, II or VI who underwent follow-up DXA after receiving ERT for 1.0 to 7.4 years showed an increase in absolute BMD values, and 3 of 5 patients who started ERT before puberty demonstrated improvements in their HAZ-adjusted BMD z scores. Improvement of BMD in MPS patients receiving ERT is probably due to multiple mechanisms, such as reduced GAG storage in the bones, increased muscle strength and endurance, and improved pulmonary function and mobility [29-34]. Our findings support DXA as a clinically useful method for monitoring BMD in MPS patients and assessing its response to ERT. Further research, however, is needed to determine which of the multiple mechanisms responsible for increased BMD are most impacted by ERT. Besides ERT, patients with MPS may be candidates for investigative trials of adjunctive agents such as bisphosphonates [17].

Our study has several limitations. There were no healthy volunteers to serve as a control group. Instead, DXA results were expressed as absolute BMD values and transformed into BMD z scores based on normative reference data for Taiwanese children that account for age and gender [16,18]. The HAZ adjusted BMD z scores that account for height were calculated for our study population using reference equations for a pediatric population from the United States that was taller (few or no subjects with height z scores < −2) and non-Black (presumably mostly Caucasian) [19]. The effect of any racial differences is unknown but it is expected to be far outweighed by the major effect of height. Our study was limited to BMD of the lumbar spine, which may not be representative of other skeletal involvement. Finally, the age range of patients in our study was quite broad (5–23.7 years) and the number of patients was too small to draw any conclusions about differences between MPS types.

## Conclusions

We found a high prevalence of osteopenia (31%) or osteoporosis (15%) by DXA in MPS patients under 19 years of age unselected for skeletal problems, and all 8 patients with MPS I, II, and VI showed an increase in absolute BMD values after receiving ERT for 1–7.4 years. DXA is a simple, accurate, and non-invasive method for measuring BMD, and we recommend screening and regularly monitoring of BMD by DXA for patients with MPS. Findings of severe bone mineral loss may identify MPS patients who are at increased risk for fracture-related morbidity and mortality and prompt appropriate counseling and intervention.

## Competing interests

The authors declare that they have no competing interests.

## Authors’ contributions

HYL performed acquisition, statistical analysis and interpretation of data, and drafting of the manuscript. SPL participated in design of the study, interpretation of the data and helped to draft the manuscript. SCS and CKC performed biochemical analyses and revised the manuscript. MRC and DMN were responsible for patient screening. All authors read and accepted the manuscript.
